# Prosthetic valve endocarditis due to *Streptococcus pneumoniae*

**DOI:** 10.1186/2193-1801-3-375

**Published:** 2014-07-22

**Authors:** Ayman Natsheh, Michal Vidberg, Reuven Friedmann, Eli Ben-Chetrit, Amos M Yinnon, Shoshana Zevin

**Affiliations:** Department of Medicine B, Shaare Zedek Medical Center, Jerusalem, Israel; Department of Medicine A, Shaare Zedek Medical Center, Jerusalem, Israel; Department of Geriatrics, Shaare Zedek Medical Center, Jerusalem, Israel; Infectious Disease Unit, Shaare Zedek Medical Center, Jerusalem, Israel; Division of Internal Medicine, Shaare Zedek Medical Center, affiliated with The Hebrew University-Hadassah Medical School, P.O. Box 3235, Jerusalem, Israel

**Keywords:** Infective endocarditis, Prosthetic valve endocarditis, *Streptococcus pneumoniae*, Mitral valve replacement

## Abstract

**Introduction:**

In the pre-antibiotic era up 10% of cases of infective endocarditis were due to *Streptococcus pneumoniae*, but this association is currently exceedingly rare.

**Case description:**

Since 1997 we have diagnosed three patients, all aged >70, with endocarditis due to *S. pneumoniae*. One of these three cases involved a prosthetic valve, another a prosthetic ring. All three patients completely recovered with antibiotic treatment only.

**Discussion and evaluation:**

During the same period there were 1694 cases of pneumococcal bacteremia, of whom 395 (23%) after age 70. Therefore, after age 70 the prevalence of endocarditis out of all cases of pneumococcal bacteremia was 0.7%. A literature review detected another 16 cases of pneumococcal PVE. The mean age of these 17 patients was 64±14; 10 were female and 7 male. In most instances, symptom duration was short, < 6 days. Valve surgery was performed in 5 cases (29%) and 13 patients (76%) survived.

**Conclusions:**

Endocarditis due to *S. pneumoniae* is rare in the antibiotic era; even in patients with prosthetic valves its course is evidently not more virulent than with other low-virulent organisms.

## Introduction

In the pre-antibiotic era up to 15% of all cases of infective endocarditis (IE) were due to *S. pneumoniae*, but currently <1%, almost all involving native valves. Recent data have demonstrated a relative increase in the incidence of prosthetic valves as a predisposing factor for IE, from ±13% in the 1970s and 1980s to presently 22-31% (Fefer et al. 2002). We describe three patients with pneumococcal endocarditis diagnosed since 1997, one of whom had prosthetic valve endocarditis (PVE) which is the focus of the current paper and review 16 similar patients, previously published (Killen et al. 1970, Bruyn et al. 1990, Ugolini et al. 1986, Aguado et al. 1993, Hanson et al. 1993, Cunningham and Sinha 1995, Lefort et al. 2000, Collazos et al. 1996, Claes et al. 2000, O'Brien et al. 2011).

### Patient #1

In March 2013, an 80-year old female patient presented because of an unexpected fall. She underwent mitral valve replacement 13 years earlier with a St Jude mechanical valve. She denied fever or any other complaint. Oral temperature was 37.6°C, mechanical heart sounds were heard, as well as a 2/6 apical systolic murmur. The physical examination was otherwise unremarkable.

Laboratory tests revealed a leucocyte count of 10.800/μL, hemoglobin 10.5 gm/dL, and normal liver and kidney function tests. Because of unexplained fever, three blood cultures were obtained, which grew *Streptococcus pneumoniae*, with a minimal inhibitory concentration (MIC) of <0.1 μg/mL. A trans-esophageal echocardiogram (TEE) revealed a 1.1 cm sized vegetation attached to the prosthetic mitral valve. In spite of the large vegetation and its presence on a mechanical valve it was decided not to operate, because of the patient's fragility. The patient was treated with intravenous ceftriaxone for six weeks, and she attained complete clinical and microbiological cure.

### Patient #2

In February 2006, an 81 year old fully alert woman was admitted because of swelling, erythema, local heat and pain in her left knee, which started several days after she fell. One year earlier she had undergone mitral valve repair because of severe mitral incompetence: after quadrangular resection annuloplasty was performed with a 30 mm ring. Physical examination revealed a temperature of 38°C, a 1/6 pan-systolic murmur at the apex, while the left knee was swollen, red and hot. The physical examination was otherwise unremarkable. The peripheral blood count was 9.400/μL, hemoglobin was 9.9 gm/dL and biochemistry was normal. *Streptococcus pneumoniae* was isolated from two blood cultures and from joint fluid; MIC was 0.02 μg/mL. The TEE demonstrated two vegetations < 1 cm in size attached to the posterior repaired mitral valve and ring. The patient received a six weeks course of ceftriaxone and completely recovered.

### Patient #3

A 74 year male patient was admitted in 1996 because of an acute febrile illness. There were no localizing symptoms and physical examination was negative except a 2/6 systolic murmur. *Streptococcus pneumoniae* (MIC = 0.01 μg/mL), was isolated from two blood cultures. A TEE indicated moderate mitral regurgitation, exactly as found in a routine echocardiogram obtained two years earlier. The patient received two weeks of intravenous penicillin and completely recovered. During the subsequent six months he developed exertional dyspnea without fever. Echocardiography showed significantly worsened mitral regurgitation, but no vegetations were detected. He underwent an uneventful valve replacement with a biological prosthesis. Routine histologic examination revealed an ulcerated mitral valve, with fibrinous vegetation and inflammatory infiltrate. The patient was treated with ceftriaxone for four weeks and attained complete cure. In retrospect, it seems this patient suffered from pneumococcal endocarditis, partially treated with two weeks of intravenous penicillin and subsequently developed latent endocarditis and worsening mitral insufficiency (Shapiro et al. 2004).

## Discussion

Pneumococcal endocarditis in the antibiotic era is rare and generally manifests acutely, similar to staphylococcal endocarditis, although rare instances of a more insidious course have been described. In several series of pneumococcal bacteremia in the antibiotic era the prevalence rate of endocarditis was reported, which ranges from 0.3%- 3.4% (Bruyn et al. 1990, Cunningham and Sinha 1995).

In our hospital 1694 patients have been diagnosed with pneumococcal bacteremia since 1997 (Table 1). During this period only three patients, all aged >70, were diagnosed with endocarditis, constituting 0.18% of all cases of pneumococcal bacteremia. Of these 1694 cases, 395 (23%) occurred after age 70. Therefore, after the latter age the prevalence of endocarditis out of all cases of pneumococcal bacteremia was 0.7%.

**Table 1:** **Patient specific episodes of** ***S. pneumoniae*** **bacteremia, by age (1997–2013)**

Year	Age groups (years)				***S. pneumoniae***/All positive	
	1-20	21-70	71-110	UA	Total (n, %)	All positive ^2^
					***S. pneumoniae*** ^1^	
1997	36	13	13*		62 (1.4)	439
1998	42	39	22		62 (0.7)	848
1999	43	33	18		94 (1.3)	708
2000	40	27	20		87 (1.7)	523
2001	41	15	22		78 (0.9)	891
2002	52	21	11	2	84 (1)	876
2003	73	12	13		98 (1.1)	888
2004	46	14	21		81 (1)	789
2005	48	34	37		119 (1.3)	891
2006	62	46	24*		132 (1.4)	951
2007	66	33	19	1	118 (1.1)	1029
2008	63	39	47	2	149 (1.5)	997
2009	89	28	31		148 (1.4)	1087
2010	54	25	24		103 (0.9)	1176
2011	60	20	30		110 (0.9)	1191
2012	45	19	31		95 (0.8)	1205
2013	11	10	12*		33 (0.3)	839
Total	871	428	395	5	1694 (1.0)	16515

UA, unavailable; each asterix* indicates one patient diagnosed that particular year with *S. pneumoniae* endocarditis.

The difference in incidence of *S. pneumoniae* endocarditis between age 1–20 (0/871), adults aged 21–70 (0/428) and elderly (3/395 or 0.8%) was statistically insignificant.

All patient-specific blood isolates of *S. pneumoniae* ^1^ (n, and as % of all patient-specific true-positive blood isolates^2^ (i.e. excluding contaminants).

One of our three pneumococcal endocarditis cases involved a prosthetic valve, another a repaired mitral valve and ring, possibly suggesting a higher propensity of *S. pneumoniae* to infect prosthetic rather than natural valves. This trend has not previously been reported: the Bruyn et al. reported five patients with pneumococcal endocarditis of whom one had PVE Bruyn et al. (1990)), and Lefort et al. (2000) reported 30 cases with pneumococcal endocarditis, collected in a nation-wide survey of whom 4 (13%) had PVE.

A literature review detected another 16 cases of PVE with this organism (Table 2). The mean age of these 17 patients was 64 ± 14; 10 were female and 7 male. In most instances, symptom duration was short, < 6 days. Valve surgery was performed in 5 cases (29%) and 13 patients (78%) survived.

**Table 2:** **Reported patients with prosthetic valve endocarditis associated with** ***Streptococcus pneumoniae***

No, [ref]	Year	Sex, age	Valve	PMH	Days of symptoms	Source of infection	Diagnostic method	Valve surgery	Outcome
1 [Killen et al. 1970]	1970	M, 24	Aortic	NA	5	NA	NA	Yes	Died
2 [Killen 1970]	1970	F, 51	Tricuspid	NA	3	NA	NA	No	Died
3 [Buyn 1990]	1982	F, 71	Mitral	CHF	NA	Lung	No vegetations	No	Cure
4 [Ugolini et al. 1986]	1986	M, 50	Aortic	DM	5	Sinusitis	Clinical suspicion	No	Cure
5 [Bruyn et al. 1990]	1990	F, 71	Mitral	None	1	Lung	Clinical suspicion	No	Cure
6 [Aguado et al. 1993]	1993	M, 53	Aortic	NA	NA	Na	NA	Yes	Cure
7 [Aguado et al. 1993, 1993]	1993	F, 74	Mitral	None	1	Dental procedure	TEE	No	Cure
8 [Cunningham and Sinha 1995]	1994	M, 63	Aortic + mitral	Previous IE	14	-	Clinical suspicion	No	Cure
9 [Lefort 2000]	1994	F, 80	Aortic + mitral	Alcoholism DM	30	Unknown	TEE	Yes	Cure
10 [Lefort et al. 2000]	1994	F, 79	Aortic + mitral	None	NA	Lung	No vegetation	No	Cure
11 [Lefort et al. 2000]	1995	M, 73	Aortic	None	21	Lung	TEE	Yes	Died
12 [Cunningham and Sinha 1995]	1995	M, 63	Mitral	None	7	Unknown	Clinical suspicion	No	Cure
13 [Lefort et al. 2000]	1996	M, 58	Mitral	Alcoholism	46	Lung	TEE	No	Died
14 [Collazos et al. 1996]	1996	F, 61	Mitral	None	6	Unknown	TEE	No	Cure
15 [Claes et al. 2000]	1999	F, 61	Aortic + mitral	Atrial fib	NA	Lung	TEE	Yes	Cure
16 [O'Brien et al. 2011]	2011	F, 63	Aortic	Previous IE	-	-	Roth spots + BCs	No	Cure
17 Present patient #1	2013	F, 80	Mitral	None	NA	Unknown	TEE	No	Cure

Atrial Fib, atrial fibrillation; BC, blood cultures; CHF, congestive heart failure; DM, diabetes mellitus; NA: data not available; PMH, past medical history; Present 1/2, Present case 1/2; TEE, transesophageal echocardiogram; Valve, infected mechanical or biological prosthetic valve. Patient no. 2 had endocarditis from both *Staphylococcus aureus* and *Streptococcus pneumoniae*.

In conclusion, in the antibiotic era endocarditis due to *Streptococcus pneumoniae* is rare. Importantly, even in patients with pneumococcal PVE its course may be insidious and not more aggressive than with other low-virulent organisms.
